# Quantitative image variables reflect the intratumoral pathologic heterogeneity of lung adenocarcinoma

**DOI:** 10.18632/oncotarget.11693

**Published:** 2016-08-30

**Authors:** E-Ryung Choi, Ho Yun Lee, Ji Yun Jeong, Yoon-La Choi, Jhingook Kim, Jungmin Bae, Kyung Soo Lee, Young Mog Shim

**Affiliations:** ^1^ Department of Radiology and Center for Imaging Science, Samsung Medical Center, Sungkyunkwan University School of Medicine, Seoul, Korea; ^2^ Department of Pathology, Kyungpook National University Hospital, Kyungpook National University School of Medicine, Daegu, Korea; ^3^ Department of Pathology, Samsung Medical Center, Sungkyunkwan University School of Medicine, Seoul, Korea; ^4^ Department of Thoracic and Cardiovascular Surgery, Samsung Medical Center, Sungkyunkwan University School of Medicine, Seoul, Korea

**Keywords:** lung adenocarcinoma, heterogeneity, radiomics, quantitative image variables, dual energy CT

## Abstract

We aimed to compare quantitative radiomic parameters from dual-energy computed tomography (DECT) of lung adenocarcinoma and pathologic complexity.

A total 89 tumors with clinical stage I/II lung adenocarcinoma were prospectively included. Fifty one radiomic features were assessed both from iodine images and non-contrast images of DECT datasets. Comprehensive histologic subtyping was evaluated with all surgically resected tumors. The degree of pathologic heterogeneity was assessed using pathologic index and the number of mixture histologic subtypes in a tumor. Radiomic parameters were correlated with pathologic index. Tumors were classified as three groups according to the number of mixture histologic subtypes and radiomic parameters were compared between the three groups.

Tumor density and 50^th^ through 97.5^th^ percentile Hounsfield units (HU) of histogram on non-contrast images showed strong correlation with the pathologic heterogeneity. Radiomic parameters including 75^th^ and 97.5^th^ percentile HU of histogram, entropy, and inertia on 1-, 2- and 3 voxel distance on non-contrast images showed incremental changes while homogeneity showed detrimental change according to the number of mixture histologic subtypes (all *Ps* < 0.05).

Radiomic variables from DECT of lung adenocarcinoma reflect pathologic intratumoral heterogeneity, which may help in the prediction of intratumoral heterogeneity of the whole tumor.

## INTRODUCTION

Lung cancer is the most commonly diagnosed cancer worldwide and the leading cause of cancer-related death [1], and adenocarcinoma is the most common histologic subtype of lung cancer in most countries [2]. In an attempt to reflect the widely divergent pathologic spectrum of lung adenocarcinoma, lung adenocarcinoma classification criteria were proposed by the International Association for the Study of Lung Cancer/American Thoracic Society/European Respiratory Society (IASLC/ATS/ERS) [3]. This classification was devised to understand the histological subtypes and their histo-molecular correlations. Since the release of this classification, many studies have investigated the possible correlations among the most predominant subtypes, driver mutations and patient prognosis [4, 5]. However, prognostic stratification considering only the most predominant subtypes has shown substantial limitation due to the fact that more than 80% of invasive lung adenocarcinomas show mixed type including two or more of histologic subtypes [6–9].

Recently, scientists have performed quantitative imaging of lung cancer using primarily a radiomic approach, demonstrating that radiomic values quantifying spatial variation in architecture have shown prognostic significance [10–12]. Based on these results, they suggested that imaging features depicting spatial heterogeneity in tumors might reflect genomic and phenotypic intratumoral heterogeneity, which has significant implications for treatment, resistance, and, ultimately, prognosis [13–15]. Surprisingly, there have not been any studies directly evaluating the relationship between radiologic heterogeneity values and pathologic complexity within lung adenocarcinoma. Thus, we aimed to correlate various quantitative radiomic parameters from dual-energy computed tomography (DECT) of lung adenocarcinomas with pathologic complexity to ultimately identify the role of quantitative image variables in predicting pathologic heterogeneity.

## RESULTS

### Clinical characteristics of patients and tumors

**Figure 1:**
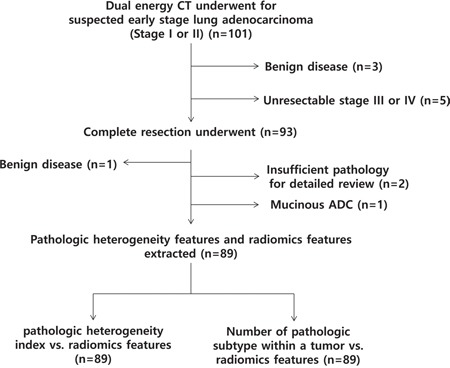
Flow diagram of the patient cohort

All 92 consecutive patients were enrolled and underwent DECT for work-up (Figure 1). We excluded three patients who were shown to have benign disease after percutaneous lesion biopsy and five patients who were found to have unresectable stage III or IV lung cancer through further studies. Eight-four patients underwent complete resection for ninety-three lesions. Of those, we excluded four who had one benign disease, one who had mucinous adenocarcinoma, and two who had insufficient pathologic slides for detailed pathologic review. In total, 80 patients with 89 lung adenocarcinomas were included in our study.

There were total 6 patients who have more than one primary lesion arising in the lung at the same time. Three patients had 2 tumors in the lung and three patients had 3 tumors in the lung. Among these patients, four had nodules in the same lobe. However, multiple tumors in the same lobe showed complete different solidity on CT scan suggesting intendent relationships more likely than the metastasis. Pathological result revealed these tumors were synchronous tumors. The most predominant histologic subtype was the acinar subtype (59.6%) followed by lepidic subtype (20.2%) (Table 1). The most frequent combinations in mixed type were lepidic and acinar subtypes (14.6%). Final pathologic staging revealed 84 stage I tumors, one stage II tumor, and four stage III tumors. The clinicopathologic characteristics of the 89 lung adenocarcinomas included in this study are summarized in Table 1. Also, the relationship between size and volume of tumors are included in Supplementary Figure S1.

**Table 1:** Clinicopathologic characteristics of lung adenocarcinoma (89 tumors from 80 patients)

Characteristics			Total N (%)	
**Sex (%)**				
Male			39 (48.8)	
Female			41 (51.2)	
**Age, range (median)**			37-78 (59)	
**Tumor size (mm), range (median)**			4-64 (21)	
**Staging**				
I			84 (94.4)	
II			1 (1.1)	
III			4 (4.5)	
**T category**				
1			73 (82.1)	
2			14 (15.7)	
3			2 (2.2)	
**N category**				
0			82 (92.1)	
1			5 (5.6)	
2			2 (2.2)	
**Extent of resection (%)**				
Segmentectomy			50 (56.2)	
Lobectomy			39 (43.8)	
**Most predominant histologic subtype**				
Lepidic			18 (20.2)	
Acinar			53 (59.6)	
Papillary			11 (12.4)	
Solid			1 (1.1)	
Micropapillary			6 (6.7)	
**No. of histologic subtypes within a tumor**				
1	19 (21.3)	Lepidic		5 (26.3)
		Acinar		11 (57.9)
		Papillary		3 (15.8)
2	54 (60.7)	Lepidic + Acinar		31 (57.4)
		Acinar + Papillary		13 (24.1)
		Acinar + Micropapillary		1 (1.9)
		Acinar + Solid		9 (16.7)
3	14 (15.7)	Lepidic + Acinar + Papillary		6 (42.9)
		Lepidic + Acinar + Solid		2 (14.3)
		Acinar + Papillary + Micropapillary		4 (28.6)
		Acinar + Micropapillary + Solid		2 (14.3)
4	2 (2.2)	Lepidic + Acinar + Papillary + Micropapillary		2 (100)

### Correlation between radiomic features and pathology heterogeneity index

There was strong relationship between the prediction model made with selected radiomic variables and pathologic heterogeneity index (*R* = 0.936, *p* = 0.001).

The relationships between 51 radiomic parameters and the pathologic heterogeneity index are described in Table 2. Among the radiomic parameters of physical features, density on non-contrast images showed a strong correlation with pathologic heterogeneity (*R* = 0.654, *P* < 0.001). Also, pathologic heterogeneity was robustly associated with 50^th^ percentile, 75^th^ percentile and 97.5^th^ percentile of HU among histogram CT parameters on non-contrast images (*R* = 0.648, *R* = 0.663, *R* = 0.626, respectively; all *Ps* < 0.001). The size of the tumor on iodine-contrast images shows moderate positive correlation (*R* = 0.527, *P* < 0.001). The skewness of histogram features showed a moderate negative correlation with histologic complexity on both non-contrast and iodine-contrast images (*R* = −0.532, *R* = −0.531, respectively; all *Ps* < 0.001). There was no strong association between regional features. Among local features, variance and inertia on non-contrast image were moderately associated with pathologic complexity (variance on 1 voxel distance, *R* = −0.522; variance on 2 voxel distance, *R* = −0.517; variance on 3 voxel distance, *R* = −0.521; inertia on 1 voxel distance, *R* = 0.549; inertia on 2 voxel distance, *R* = 0.550; inertia on 3 voxel distance, *R* = 0.574; all *Ps* < 0.001).

**Table 2:** Correlation of radiomic features with pathologic heterogeneity index

CT parameters	Non-contrast images		Iodine-contrast images	
	R	P	R	P
**Physical features**				
Volume (cm^3^)	0.233	0.028*	0.177	0.096
Density (HU)	0.653**	< 0.001*	0.139	0.195
Mass (g)	0.352	0.001*	0.183	0.086
Size (mm)	0.252	0.017	0.527**	< 0.001*
**Histogram features**				
Skewness	-0.532**	< 0.001*	-0.531**	< 0.001*
Kurtosis	−0.059	0.584	−0.313	0.003*
2.5th percentile (HU)	0.424	< 0.001*	−0.423	< 0.001*
25th percentile (HU)	0.584**	< 0.001*	0.099	0.356
50th percentile (HU)	0.648**	< 0.001*	0.254	0.016*
75th percentile (HU)	0.663**	< 0.001*	0.246	0.020*
97.5th percentile (HU)	0.626**	< 0.001*	−0.180	0.091
**Regional features**				
Uniformity	−0.195	0.067	−0.140	0.190
Entropy	0.297	0.005*	0.131	0.223
Intensity variability	0.276	0.009*	0.368	< 0.001*
Size-zone variability	0.245	0.021*	0.171	0.108
**Local features**				
Energy on 1 voxel distance	0.311	0.003*	−0.073	0.496
Energy on 2 voxel distance	0.249	0.020*	−0.060	0.579
Energy on 3 voxel distance	0.184	0.087	−0.134	0.211
Entropy on 1 voxel distance	−0.192	0.073	0.132	0.219
Entropy on 2 voxel distance	−0.112	0.298	0.117	0.276
Entropy on 3 voxel distance	−0.065	0.550	0.193	0.070
Correlation on 1 voxel distance	−0.127	0.239	−0.100	0.350
Correlation on 2 voxel distance	0.009	0.934	0.029	0.791
Correlation on 3 voxel distance	0.112	0.299	0.063	0.559
Contrast on 1 voxel distance	−0.032	0.770	0.061	0.568
Contrast on 2 voxel distance	0.012	0.914	−0.028	0.793
Contrast on 3 voxel distance	0.073	0.500	−0.039	0.716
Variance on 1 voxel distance	-0.522**	< 0.001*	−0.385	< 0.001*
Variance on 2 voxel distance	-0.517**	< 0.001*	−0.382	< 0.001*
Variance on 3 voxel distance	-0.521**	< 0.001*	−0.409	< 0.001*
Sum mean on 1 voxel distance	0.238	0.025*	0.027	0.804
Sum mean on 2 voxel distance	0.222	0.038*	−0.014	0.9
Sum mean on 3 voxel distance	0.235	0.027*	−0.017	0.875
Inertia on 1 voxel distance	0.549**	< 0.001*	0.391	< 0.001*
Inertia on 2 voxel distance	0.550**	< 0.001*	0.384	< 0.001*
Inertia on 3 voxel distance	0.574**	< 0.001*	0.401	< 0.001*
Cluster shade on 1 voxel distance	−0.032	0.770	0.061	0.568
Cluster shade on 2 voxel distance	0.012	0.914	−0.028	0.793
Cluster shade on 3 voxel distance	0.073	0.500	−0.039	0.716
Cluster tendency on 1 voxel distance	−0.454	< 0.001*	−0.434	< 0.001*
Cluster tendency on 2 voxel distance	−0.438	< 0.001*	−0.420	< 0.001*
Cluster tendency on 3 voxel distance	−0.448	< 0.001*	−0.470	< 0.001*
Homogeneity on 1 voxel distance	0.406	< 0.001*	−0.207	0.052
Homogeneity on 2 voxel distance	0.398	< 0.001*	−0.129	0.230
Homogeneity on 3 voxel distance	0.411	< 0.001*	−0.109	0.308
Maximum probability on 1 voxel distance	0.447	< 0.001*	−0.041	0.700
Maximum probability on 2 voxel distance	0.403	< 0.001*	−0.042	0.698
Maximum probability on 3 voxel distance	0.323	0.002*	−0.090	0.400
Inverse variance on 1 voxel distance	−0.336	0.001*	−0.102	0.343
Inverse variance on 2 voxel distance	−0.124	0.250	−0.005	0.963
Inverse variance on 3 voxel distance	−0.048	0.659	−0.023	0.833

R indicates the Spearman correlation coefficient.

* P < 0.05

** R > 0.5

### Comparison of three different groups based on pathologic heterogeneity and radiomic parameters

Among histogram features on non-contrast CT images, the 75^th^ percentile and 97.5^th^ percentile HU of the histogram showed significantly increased mean CT values according to the number of histologic subtypes (*P* = 0.003, *P* = 0.002, respectively) (Table 3).

**Table 3:** Radiomic features according to the number of histologic subtypes within a tumor

	Non-contrast images			*p*	Iodine-contrast images			*p*
	One subtype	Two subtypes	Three or four subtypes		One subtype	Two subtypes	Three or four subtypes	
**Physical features**								
Volume (cm^3^)	6.97±2.14	8.29±2.81	7.93±5.900	0.966	7.08±12.62	4.67±5.85	6.50±5.10	0.432
Density (HU)	0.57±0.23	0.566±0.22	0.75±0.155	0.008*	1.17±0.016	1.16±0.019	1.17±0.01	0.618
Mass (g)	5.30±10.35	4.572±9.00	6.00±4.484	0.832	8.25±14.71	5.44±6.78	7.61±5.95	0.426
Size (mm)	23.11±16.86	21.02±9.26	24.41±10.89	0.488	12.44±18.26	9.83±12.02	17.18±8.85	0.130
**Histogram features**								
Skewness	−0.21±0.66	0.012±0.721	−0.489±0.616	0.037*	0.461±0.822	0.782±1.04	0.056±0.598	0.024*
Kurtosis	2.75±0.96	2.76±1.01	2.47±1.108	0.598	4.39±2.32	5.60±4.33	4.51±3.29	0.377
2.5^th^ percentile (HU)	−797.76±71.95	−814.99±70.14	−769.28 ±74.37	0.080	**-21.07±14.38**	**-23.42±16.68**	**-31.39 ±14.34**	0.131
25^th^ percentile (HU)	−576.08±193.86	−604.91±181.72	−484.64 ±181.10	0.078	25.31±13.29	21.15±16.143	26.20±15.66	0.397
50^th^ percentile (HU)	−431.21±232.06	−450.78±234.06	−251.37 ±171.56	0.009*	52.50±16.20	47.94±19.68	56.62±5.35	0.220
75^th^ percentile (HU)	**-316.05±241.84**	**-305.18±232.75**	**-91.90±127.34**	0.003*	79.94±17.36	76.38±22.41	83.68±16.05	0.429
97.5^th^ percentile (HU)	**-181.87±241.84**	**-128.25±189.22**	**32.48±39.52**	0.002*	152.31±39.92	156.08±35.30	143.65±27.89	0.464
**Regional features**								
Uniformity	0.003±0.002	0.002±0.001	0.002±0.001	0.042*	0.009±0.002	0.009±0.002	0.008±0.002	0.365
Entropy	**8.76±0.73**	**9.08±0.50**	**9.41±0.37**	0.003*	**7.17±0.37**	**7.24±0.32**	**7.36± 0.31**	0.258
Intensity variability	**7.56±3.71**	**8.49±3.8**	**9.57±3.53**	0.294	7.27±3.18	6.1563.29	8.03±3.27	0.099
Size-zone variability	**13.14±12.21**	**15.33±11.86**	**18.59±7.81**	0.367	14.42±14.94	14.22±9.44	17.99±8.60	0.454
**Local features**								
Energy on 1 voxel distance	0.040±0.050	0.034±0.033	0.051±0.067	0.410	**0.037±0.017**	**0.046±0.030**	**0.049±0.036**	0.394
Energy on 2 voxel distance	0.036±0.046	0.027±0.028	0.042± 0.064	0.356	**0.033±0.014**	**0.040±0.025**	**0.043± 0.033**	0.493
Energy on 3 voxel distance	0.031±0.025	0.024±0.026	0.039± 0.061	0.337	**0.036±0.014**	**0.040±0.025**	**0.042± 0.033**	0.784
Entropy on 1 voxel distance	1.74±0.23	1.79±0.19	1.76± 0.24	0.580	**1.65±0.15**	**1.62±0.18**	**1.59±0.22**	0.697
Entropy on 2 voxel distance	1.82±0.24	1.91±0.20	1.89±0.26	0.306	1.66±0.13	1.66±0.17	1.63± 0.22	0.869
Entropy on 3 voxel distance	1.81±0.22	1.93±0.22	1.92± 0.28	0.128	1.62±0.15	1.65±0.16	1.63±0.22	0.798
Correlation on 1 voxel distance	0.089±0.037	0.091±0.046	0.079± 0.036	0.658	**0.109±0.062**	**0.121±0.059**	**0.154±0.14**	0.231
Correlation on 2 voxel distance	**0.047±0.030**	**0.053±0.024**	**0.056±0.027**	0.555	**0.034±0.027**	**0.036±0.023**	**0.052±0.057**	0.190
Correlation on 3 voxel distance	**0.025±0.038**	**0.030±0.019**	**0.040± 0.020**	0.231	0.021±0.019	0.017±0.015	0.027±0.030	0.203
Contrast on 1 voxel distance	**5.30±2.23**	**4.76±2.04**	**4.47±1.90**	0.478	**5.51±2.83**	**4.98±3.13**	**4.32±2.06**	0.492
Contrast on 2 voxel distance	11.16±4.60	10.45±4.24	10.62±5.01	0.834	**8.13±4.25**	**7.29±3.87**	**6.09± 2.88**	0.290
Contrast on 3 voxel distance	14.71±6.75	14.29±5.67	15.48± 8.17	0.807	**8.62±4.36**	**7.94±4.19**	**6.38± 2.86**	0.248
Variance on 1 voxel distance	**0.17±0.11**	**0.163±0.054**	**0.129± 0.029**	0.114	0.176±0.053	0.185±0.052	0.175±0.054	0.704
Variance on 2 voxel distance	**0.17±0.10**	**0.161±0.054**	**0.129± 0.032**	0.121	0.177±0.052	0.186±0.051	0.177± 0.056	0.718
Variance on 3 voxel distance	0.16±0.06	0.162±0.054	0.131± 0.038	0.111	0.179±0.050	0.188±0.051	0.179± 0.058	0.765
Sum mean on 1 voxel distance	**8.83±3.47**	**9.60±3.41**	**11.544±3.509**	0.061	**4.37±4.05**	**4.05±1.92**	**3.48±1.43**	0.351
Sum mean on 2 voxel distance	**8.90±3.51**	**9.68±3.48**	**11.354± 3.407**	0.111	**4.37±1.92**	**4.02±1.87**	**3.40±1.46**	0.285
Sum mean on 3 voxel distance	**8.46±3.06**	**9.79±3.57**	**11.369± 3.468**	0.051	**4.27±1.75**	**4.02±1.77**	**3.36± 1.44**	0.267
Inertia on 1 voxel distance	**8.37±2.23**	**8.43±2.28**	**10.057± 1.532**	0.027*	6.87±1.71	6.50±1.64	6.85±1.71	0.617
Inertia on 2 voxel distance	**8.45±2.26**	**8.54±2.31**	**10.20±1.54**	0.024*	6.82±1.66	6.47±1.60	6.82± 1.70	0.621
Inertia on 3 voxel distance	**8.39±2.26**	**8.54±2.31**	**10.247± 1.554**	0.018*	6.70±1.56	6.44±1.56	6.78± 1.69	0.672
Cluster shade on 1 voxel distance	**5.30±2.23**	**4.76±2.04**	**4.479±1.903**	0.478	**5.51±2.83**	**4.98±3.13**	**4.33±2.07**	0.492
Cluster shade on 2 voxel distance	11.16±4.60	10.43±4.24	10.629±5.011	0.834	**8.14±4.26**	**7.30±5.68**	**6.10±2.88**	0.290
Cluster shade on 3 voxel distance	14.71±6.75	14.29±5.67	15.480± 8.177	0.807	**8.62±4.37**	**7.941±3.87**	**6.38± 2.86**	0.248
Cluster tendency on 1 voxel distance	−43.74±16.58	−42.39±50.95	−108.582±29.33	0.244	14.25±29.92	14.76±27.81	3.07±20.57	0.309
Cluster tendency on 2 voxel distance	**-37.71±86.04**	**-36.77±116.02**	**-82.633± 04.72**	0.318	7.57±18.33	8.30±16.88	1.58±15.04	0.374
Cluster tendency on 3 voxel distance	−33.78±64.46	−32.49±95.11	−70.196±88.10	0.314	**7.34±15.93**	**6.95±13.80**	**1.56± 14.53**	0.388
Homogeneity on 1 voxel distance	**4532.07±1777.15**	**3387.64±2166.78**	**2629.69±181.00**	0.025*	**629.72±389.37**	**565.77±348.42**	**386.11± 232.51**	0.094
Homogeneity on 2 voxel distance	**3480.25± 1460.08**	**2523.03±1782.39**	**1899.41±1225.18**	0.023*	**375.41±229.52**	**341.68±234.28**	**243.52± 173.64**	0.197
Homogeneity on 3 voxel distance	**2895.88±1303.93**	**2039.34±1521.84**	**1380.43±1060.43**	0.008*	**314.92±192.11**	**289.38±191.10**	**215.53± 177.30**	0.273
Maximum probability on 1 voxel distance	0.109±0.110	0.092±0.079	0.137± 0.125	0.248	**0.085±0.041**	**0.110±0.064**	**0.116± 0.067**	0.230
Maximum probability on 2 voxel distance	0.095±0.102	0.072±0.071	0.117± 0.123	0.189	**0.076±0.033**	**0.095±0.055**	**0.101± 0.062**	0.308
Maximum probability on 3 voxel distance	0.081±0.0619	0.064±0.065	0.107± 0.120	0.154	**0.078±0.029**	**0.092±0.051**	**0.096± 0.061**	0.491
Inverse variance on 1 voxel distance	0.381±0.063	0.402±0.045	0.381± 0.051	0.153	0.413±0.050	0.417±0.045	0.416±0.031	0.952
Inverse variance on 2 voxel distance	0.316±0.060	0.339±0.049	0.322± 0.047	0.185	**0.377±0.066**	**0.386±0.054**	**0.396±0.046**	0.598
Inverse variance on 3 voxel distance	0.294±0.055	0.305±0.051	0.289± 0.049	0.487	**0.372±0.059**	**0.375±0.056**	**0.390±0.047**	0.558

Features showing uniform tendency are expressed in bold font.

* P < 0.05

Of all regional features, entropy showed an escalation of the mean CT values with an increased number of histologic subtypes on non-contrast images with statistical significance (*P* = 0.003). Although those were not statistically significant, intensity variability and size-zone variability on the non-contrast images and entropy on the iodine images presented increased mean CT values according to the number of histologic subtypes.

Among local features on non-contrast images, the mean CT values of inertia increased while those of homogeneity demonstrate a declined as the number of histologic subtypes increased with statistical significance (Inertia on 1 voxel distance, *P* = 0.027; Inertia on 2 voxel distance, *P* = 0.024; Inertia on 3 voxel distance, *P* = 0.018; homogeneity on 1 voxel distance, *P* = 025; homogeneity on 2 voxel distance, *P* = 023; homogeneity on 3 voxel distance, *P* = 0.008). On iodine-enhanced images, the mean CT values of energy and maximum probability value increased while those of contrast, sum mean, cluster shade, and homogeneity showed a decline when the number of histologic subtypes increased. However, the tendencies of these values were failed to show the statistical significance.

All radiomic parameters of the three different groups based on pathologic heterogeneity are described in Table 3.

## DISCUSSION

Recent lung cancer research has demonstrated that cancerous cells not only undergo clonal evolution from a single progenitor, but also exhibit branched evolution, whereby each tumor develops and preserves multiple distinct subclonal compositions [16–19]. This intratumoral genetic heterogeneity consequently leads to phenotypic differences in histopathological divergence, containing regions demarcated by various degrees of differentiation, proliferation, vascularity, and invasiveness [20]. Given the existence of such heterogeneity in tumors in advanced metastatic disease, the efficacy of therapies targeting somatic driver aberrations may be limited [13]. Recent oncology research has mostly focused on the intratumoral variation in gene mutation and expression, whereas few studies have explored the spatial relationship among imaging, genomics, and histopathology.

While imaging is a main tool in tumor staging, progression assessment and recurrence detection, most radiologic approaches deal only with tumor size or average parameter values [12] in routine oncologic practice and research. Considerable effort has explored sophisticated and robust analyses to quantify tumor spatial complexity with tumor imaging data [10, 21–25]. This approach usually uses a texture analysis method to quantify the spatial variation as the remaining spatial arrangement of voxel values in imaging data [12], which could serve as a potential prognostic biomarker [11, 26]. Most studies of radiomic analyses have evaluated the role of radiomics in the prognostic stratification [12], but there have not yet been any studies correlating intratumoral heterogeneity on CT images with pathology heterogeneity. Radiomic analysis is emerging as a method to quantify spatial variation within tumors. Establishment of a histologic correlation would obviously be an important step in the validation of this approach. Our study is the first study to determine a direct relationship between the histological complexity of lung adenocarcinoma and intratumoral heterogeneity expressed as CT radiomic values.

We adopted histogram, local and regional features to measuring degree of intratumoral heterogeneity. Histogram features were derived from the density distribution of the tumor reflecting heterogeneity of intratumoral density. The spatial arrangement of voxel values is obtained from local features. We included tumor size as a routine practical measurement variable and compared with other all radiomic variables in the analysis. Radiomic variables were more strongly correlated with pathologic heterogeneity rather than tumor size. In addition, these radiomic features may offer minimal inter- and intra-reader variability with high reproducibility of imaging features.

Our results clearly demonstrate histopathologic complexity correlated with radiomic parameters such as texture features within CT images of lung adenocarcinoma. Quantifying the spatial complexity of tumor images can explain the spatial complexity in pathology, or in other words, the phenotypic intratumoral heterogeneity.

Radiomic features derived from non-contrast images reflect tumor cellularity and density, whereas iodine-enhanced image even allows additional information about tumor vascularity. Therefore, we originally expected that radiomic features of iodine-contrast images might have associated more strongly to the tumor complexity related with heterogeneity of tumor density and tumor vascularity. However, in our analysis, the most of radiomic features which correlated to pathologic heterogeneity index were features derived from non-contrast image, not from iodine-enhanced image. This result can be explained in part by the concept of tumor microenvironment where whole tumor volume consists of real tumor component as well as nontumorous stromal component. Thus, radiomic variables extracted from non-contrast CT covering completely density or cellularity of ROI of the tumor may reflect more actually spatial heterogeneity in the entire tumor ROI, compared to radiomic variables from iodine image limitedly enhancing heterogeneity of only tumorous component. The problem is that results from small biopsy tissues in non-resectable lung adenocarcinomas do not represent the pathologic heterogeneity of the whole tumor. On the other hand, quantifying imaging data using a radiomic approach indirectly enables assessment of whole tumor heterogeneity. Quantifying spatial heterogeneity on a tumor image may help with determining the prognosis and stratifying patients with non-resectable lung adenocarcinoma for which whole tumor pathology is not available. Furthermore, intratumoral heterogeneity may have important consequences for personalized medicine approaches that typically rely on a single tumor-biopsy to portray the tumor mutational landscape. Quantitative radiomic variables could allow physicians to deliver more optimized, patient-specific treatment by allowing them to identify patients who need aggressive treatment.

In addition, radiomic parameters might be useful for noninvasive *in vivo* monitoring of longitudinal changes in tumor heterogeneity. Lung cancer cells within tumors show homogeneous cell populations until relatively late in tumor progression, when hyperproliferation and increased genetic instability result in distinct clonal subpopulations [18, 19]. Several studies have shown that mechanisms of acquired drug resistance to EGFR inhibitors are related to several genotypic and phenotypic changes [27, 28]. Using a radiogenomic approach to quantify tumor heterogeneity on images may allow longitudinal studies of clonal evolution during treatment as well.

Our study had several limitations. First, the proportion of the tumor with three or four mixtures of histologic subtypes was relatively small. The relationship of the quantitative radiomic variables and pathologic heterogeneity and the values of the quantitative radiomic variables may be influenced by the sample size. Second, all cases were collected from a single institute. Larger prospective studies from multiple centers are needed. Third, we excluded patients with variant subtypes including mucinous pattern. However, we decided to exclude this subtype because reports regarding the survival of mucinous lung adenocarcinomas are limited [29, 30].

In conclusion, various radiomic variables from DECT of lung adenocarcinoma reflect pathologic intratumoral heterogeneity, which may be a helpful predictor of intratumoral heterogeneity of the whole tumor, considering that current genomic analyses are limited by the fact that they rely on a single tumor biopsy.

## MATERIALS AND METHODS

### Patients

This study was performed as part of an ongoing prospective clinical trial aimed at determining the value of imaging biomarkers for the prediction of tumor aggressiveness and prognosis in patient with operable lung adenocarcinoma (NCT01482585). This study was approved by the institutional review board (SMC 2011-09-083) and written informed consent was obtained.

From November 2011 to December 2012, a total of 92 patients with operable lung adenocarcinoma were eligible for our study. The inclusion criteria of our study were as follows: (1) Clinically and radiologically suspected lung adenocarcinoma, (2) Newly diagnosed stage I or II disease from clinical work-up including F-18-fluorodeoxyglucose (FDG) positron emission tomography (PET)/CT, (3) Eastern Cooperative Oncology Group (ECOG) performance status of 0 or 1 and eligible for surgery, (4) Age 20 years or older, (5) Able to tolerate DECT imaging as required per protocol, and (6) Able to give study-specific informed consent. The exclusion criteria were: (1) Prior malignancy, (2) Scheduled for definitive radiation therapy or neoadjuvant concurrent chemoradiation therapy, and (3) Poor cardiopulmonary reserve (a contraindication for surgery).

### CT imaging and analysis

Patients underwent CT examination using a dual-source CT scanner (Somatom Definition Flash; Siemens Medical Solutions, Forchheim, Germany) with the dual-energy technique. Three types of data set were generated from the DECT scanning: 80 kV, 140 kV, and enhanced weighted-average images. See the supplement and Supplementary Figure S2 for further details about the image acquisition protocol and reconstruction process.

Virtual non-enhanced images and iodine-enhanced images were generated using the liver Virtual Non-Contrast (VNC) application node of dedicated dual-energy post-processing software (Syngo Dual Energy; Siemens Medical Solution, Forchheim, Germany). To obtain the iodine value of both the solid and ground-glass opacity (GGO) component in each tumor, post-processing was performed with two different types of software. Image data were reconstructed with a section thickness of 1 mm using a D30f (medium smooth) kernel for the iodine-enhanced image and a D45f (medium smooth) kernel for the virtual non-enhanced image.

In the quantitative analysis, regions of interest (ROIs) were delineated on the axial images to generate a volume of interest which included the entire tumor. Initially, we assessed the stability of all 51 radiomic parameters for which we performed the concordance correlation coefficients (CCC) regarding radiomic values extracted from two ROIs drawn by two radiologists in 25 randomly selected patients. From the stability test, we found that all radiomic parameters were stable, where all CCCs were very high or high (range, 0.888 – 0.999; mean, 0.934; SD, 0.008). As a next step, all included patient were handled by one radiologist.

**Figure 2:**
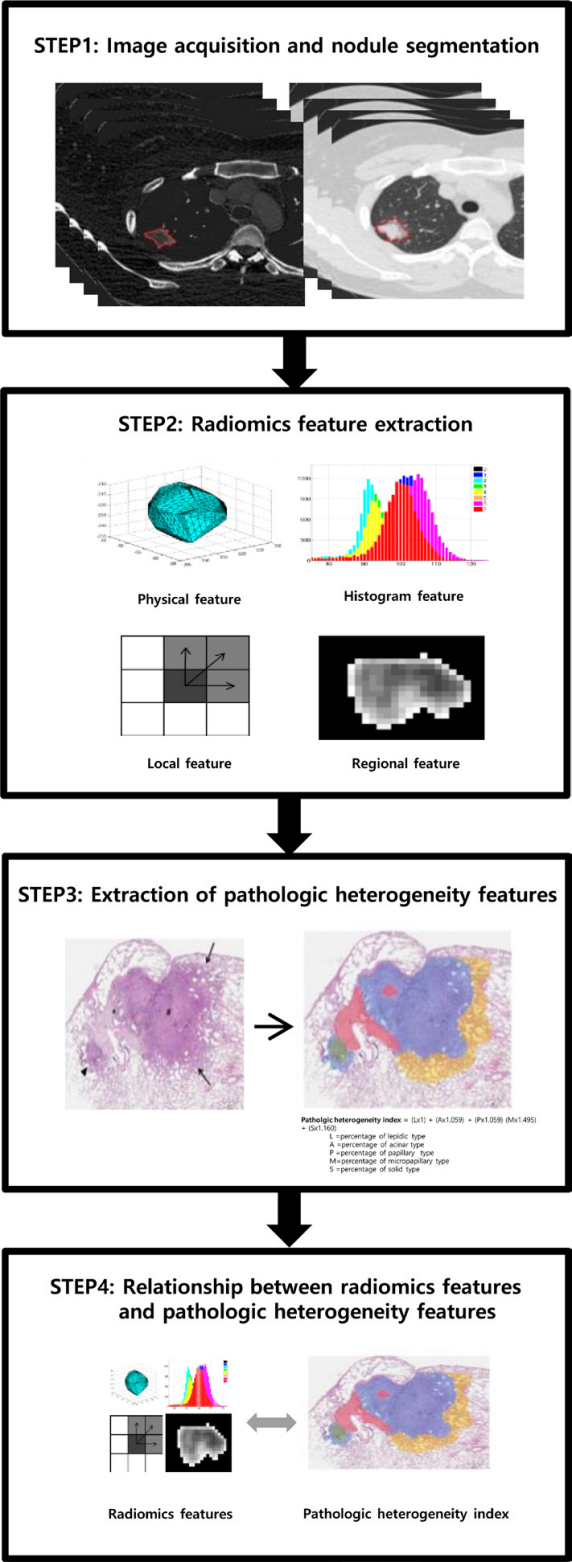
Radiomic data extraction and analysis workflow

Fifty one quantitative radiomic features were derived from this ROI to evaluate the heterogeneity of the tumors (Figure 2). The physical features included volume, size, density and mass. The histogram features included skewness, kurtosis, and Hounsfield units (HU) at the 2.5^th^, 25^th^, 50^th^, 75^th^ and 97.5^th^ percentiles. The regional features included uniformity, entropy, intensity variability and size-zone variability. There were 36 local texture features, including energy, entropy, correlation, contrast, variance, sum mean, inertia, cluster shade, cluster tendency, homogeneity, maximum probability, and inverse variance. Each local texture feature was derived from 13 directions according to 1-voxel, 2-voxel, and 3-voxel distances at the gray value for each voxel. These radiomic features were evaluated both on non-contrast images and iodine contrast images. See the supplement for further details regarding the extraction of quantitative radiomic parameters.

### Pathologic evaluation and analysis

For tumor sampling, tumor tissues with an interval of per approximately 10 mm were taken from the tumor specimen and placed on a slide. All slides were scanned to produce a high- resolution digital image (0.25 μm/pixel at 40× magnification) using the Aperio Slide Scanning System (ScanScope T3; Aperio Technologies Inc., Vista, CA, USA). Two experienced lung pathologists with 13 and 18 years of experience (J.Y.J., Y.C.) interpreted all tissue sections by virtual slides using ImageScope viewing software (Aperio Technologies, Inc.) and a high-resolution monitor. For each case, the specimens were reviewed according to the International Association for the Study of Lung Cancer (IASLC), the American Thoracic Society (ATS), and the European Respiratory Society (ERS) International Multidisciplinary Lung Adenocarcinoma Classification Criteria [3] and comprehensive histologic subtyping was performed for a whole primary tumor in a semi-quantitative manner. The extent of existent tumor histologic subtypes and central fibrosis was quantified to the nearest 5% level, adding up to a total of 100% of the subtype components per tumor. They reported each of tumors as a relative ratio among total 100% in terms of all five with its whole histologic subtype.

The pathologic heterogeneity index was calculated from each tumor to evaluate the degree of pathologic heterogeneity, which was confirmed to reflect the survival predictive value according to the proportion of mixed histologic subtypes that were derived from the hazard ratio (HR) of each subtype using the disease-free survival curve of a large-scale study [4]. Further details about the pathologic heterogeneity index are described in the supplement.

### Statistical analysis

A multivariate logistic regression model with the stepwise variable selection procedure based on Akaike's Information Criterion (AIC) was applied to validate the performance of selected radiomic features to predict actually pathologic heterogeneity. Ten-fold cross-validation [31] was used to evaluate the performance of this prediction model. After then, Pearson correlation analysis was applied to compare the predictive values from made prediction model with pathologic heterogeneity in each. The Spearman correlation coefficient was used to determine the relationship between radiomic features and pathologic heterogeneity index. When statistically significant, an absolute Rho (ρ) value between 0.00 and 0.19 was considered very weak correlation, 0.20-0.39, weak, 0.40–0.59, moderate, 0.60-0.79 strong and greater than 0.80, strong [32]. Radiomic parameters were compared among the three different groups that had been classified according to the number of histologic subtype within a tumor (e.g., tumor consistent with a single histologic subtype, two histologic subtypes, or three or four histologic subtypes) using ANOVA with Tukey's post hoc test. In the cases with multiple tumors in a patient, we did not take into account within-patient correlation because each tumor was considered an independent synchronous lesion [33].

Statistical significance was evaluated with software (SPSS, version 19.0, 2010; SPSS, Chicago, Ill). A P value less than 0.05 was considered to represent a statistically significant difference.

## SUPPLEMENTARY MATERIAL FIGURES AND TABLE




