# Supplementary material for: Quantitative image variables reflect the intratumoral pathologic heterogeneity of lung adenocarcinoma
Source: Oncotarget. 2016 Aug 30;7(41):67302–13. doi: 10.18632/oncotarget.11693 (PMC5341876; doi:10.18632/oncotarget.11693)
Supplement: Supplementary file 1 [file oncotarget-07-67302-s001.pdf]

Quantitative image variables reflect the intratumoral pathologic heterogeneity of lung adenocarcinoma

SUPPLEMENTARY MATERIALS, FIGURES AND TABLE

Imaging and analysis

All patients underwent CT examination using a dual-source CT system (Somatom Definition Flash; Siemens Healthcare, Forchheim, Germany) with the dual-energy technique (Supplementary Figure S2). The DECT system was composed of two X-ray tubes and two corresponding 128-row detectors mounted in a perpendicular arrangement. DECT scanning was obtained 90 seconds after administration of contrast material (100 mL of iopamidol: Iomeron 300; Bracco, Milan, Italy) at a rate of 1.5 mL/sec using a power injector. This was followed by a 20-cc saline flush at a rate of 1.5 mL/sec. Imaging parameters were as follows: 105 mAs (effective) at 140 kV, 248 mAs (effective) at 80 kV, 32×0.6-mm collimation, pitch of 0.7, rotation time of 0.5 seconds, and a 512×512-pixel matrix and an in-plain resolution of 0.69mm. Imaging was performed from the thoracic inlet to the middle portion of the kidneys. Three different data sets were generated from the DECT imaging: 80-kV, 140-kV, and enhanced weighted-average images. The weighted-average images were generated by combining the 140-kV and 80-kV data sets with a weighting factor of 0.6 (60% of information derived from the 80-kV image and 40% derived from the 140-kV image), and thus these were approximately 120-kV images.

Data post-processing and image reconstruction

The virtual non-enhanced images and iodine-enhanced images were generated using the liver virtual non-contrast (VNC) application mode of dedicated dual-energy post-processing software (Syngo Dual Energy; Siemens Medical Solutions) (Supplementary Figure S1). To obtain the iodine value of both solid and ground-glass opacity (GGO) components in each tumor, post-processing was performed with two different software applications. For the solid component, the parameters for the material decomposition method were as follows: -110 Hounsfield units (HU) for fat at 80 kV, -87 HU for fat at 140 kV, 52 HU for soft tissue at 80 kV, and 51 HU for soft tissue at 140 kV. For the GGO component, since the lesion was composed of a mixture of air and soft tissue, the HU value of fat in the liver VNC application mode should be replaced with that of air, which is a HU value located at the interconnecting line between air and soft tissue[1, 2].

Thus, the material parameters were -110 HU for fat at 80 kV, -115 HU for fat at 140 kV, 60 HU for soft tissue at 80 kV, and 54 HU for soft tissue at 140 kV. Image data were reconstructed with a section thickness of 1 mm using a D30f (medium smooth) kernel for the iodine-enhanced image and a D45f (medium sharp) kernel for the virtual non-enhanced image.

Quantitative CT image parameters

We adopted radiomic features to extract multi-dimensional information of a given ROI (Supplementary Table S1) [3, 4]. For physical features, the volume of the ROI was computed by multiplying the number of voxels by the unit volume of a voxel. Physical density (g/cm^3) was estimated by extrapolating from the mean CT scan attenuation [5]. Next, histogram features were derived from the intensity (HU) distribution of a given region of interest (ROI). From the histogram, we computed skewness, kurtosis, uniformity, entropy, and CT numbers or HU at the 2.5th, 25th, 50th, 75th and 97.5th percentiles. For regional features, the ROI was computed based on the gray level size zone matrix. The value of the matrix (m, n) was defined by the number of homogenous regions given the homogeneous tumor size (n) to their intensity (m). The intensity variability and size-zone variability were also computed from the gray level size zone matrix [5-9]. For local features, a gray level co-occurrence matrix (GLCM) [10], which describes the frequency of various combinations of grey values within an ROI, was created. For each ROI, GLCMs were created with 13 directions according to 1-voxel, 2-voxel, and 3-voxel distances (3 GLCMs for each ROI). From each GLCM, 12 local features (the energy, entropy, correlation, contrast, variance, sum mean, inertia, cluster shade, cluster tendency, homogeneity, maximum probability, and inverse variance) were computed. In summary, we extracted four physical, nine histogram-based, two regional, and 36 local features from the manually derived ROI.

Pathologic index

The individual prognostic impact of each subtype of lung adenocarcinoma was assessed from the development cohort using the disease-free survival (DFS) curve of a previous large-scale study [11]. Based on the

calculated hazard ratio (HR) of each subtype, four indices representing pathologic heterogeneity were developed. The first index (pathologic index 1) was defined as the sum of the proportions of each subtype multiplied by their HRs, with the addition of entropy. Entropy was calculated as: $\text{Entropy} = -\sum(p_i)\ln(p_i)$, where (p_i) represents the proportion of subtypes in the tumor [sum of all (p_i) = 1] [12]. A higher entropy score represents increased heterogeneity of the tumor. The second pathologic index (pathologic index 2) was defined as the sum of the proportions of each subtype multiplied by their HRs, with the addition of the Gini coefficient. The Gini coefficient was calculated using the following equation: $\text{Gini coefficient} = 1 - \sum(p_i^2)$. The third pathologic index (pathologic index 3) was calculated as the sum of all subtype percentages multiplied by their HRs. the fourth pathologic index (pathologic index 4) was defined as the simple arithmetic sum of the scores of the subtypes multiplied by their HRs.

These four pathologic indices were used with a validation group of 148 patients with comprehensive histologic subtyping for completely resected lung adenocarcinomas. DFS curves were plotted and the predictive ability of each pathologic index was evaluated. Among the four pathologic indices, only pathologic index 3 enabled significant patient stratification in the validation cohort according to DFS (P = 0.005). Furthermore, pathologic index 3 showed the highest concordance probability estimate of 0.691 (P = 0.049; 95% CI: 0.633-0.749) of all four pathologic indices. Thus, we used index 3 as our pathologic heterogeneity index.

REFERENCES

1. Chae EJ, Song JW, Seo JB, Krauss B, Jang YM, Song KS. Clinical utility of dual-energy CT in the evaluation of solitary pulmonary nodules: initial experience. Radiology. 2008; 249:671-681.
2. Gupta R, Phan CM, Leidecker C, Brady TJ, Hirsch JA, Nogueira RG, Yoo AJ. Evaluation of dual-energy CT for differentiating intracerebral hemorrhage from iodinated contrast material staining. Radiology. 2010; 257:205-211.
3. Aerts HJ, Velazquez ER, Leijenaar RT, Parmar C, Grossmann P, Carvalho S, Bussink J, Monshouwer R, Haibe-Kains B, Rietveld D, Hoebers F, Rietbergen MM, Leemans CR, et al. Decoding tumour phenotype by noninvasive imaging using a quantitative radiomics approach. Nat Commun. 2014; 5:4006.
4. Yoon HJ, Sohn I, Cho JH, Lee HY, Kim JH, Choi YL, Kim H, Lee G, Lee KS, Kim J. Decoding Tumor Phenotypes for ALK, ROS1, and RET Fusions in Lung Adenocarcinoma Using a Radiomics Approach. Medicine (Baltimore). 2015; 94:e1753.
5. de Hoop B, Gietema H, van de Vorst S, Murphy K, van Klaveren RJ, Prokop M. Pulmonary ground-glass nodules: increase in mass as an early indicator of growth. Radiology. 2010; 255:199-206.
6. Thibault G, Fertil B, Navarro C, Pereira S, Cau P, Levy N, Sequeira J, Mari J. Texture Indexes and Gray Level Size Zone Matrix Application to Cell Nuclei Classification. 2009.
7. Lee HY, Jeong JY, Lee KS, Kim HJ, Han J, Kim BT, Kim J, Shim YM, Kim JH, Song I. Solitary pulmonary nodular lung adenocarcinoma: correlation of histopathologic scoring and patient survival with imaging biomarkers. Radiology. 2012; 264:884-893.
8. Chong Y, Kim JH, Lee HY, Ahn YC, Lee KS, Ahn MJ, Kim J, Shim YM, Han J, Choi YL. Quantitative CT variables enabling response prediction in neoadjuvant therapy with EGFR-TKIs: are they different from those in neoadjuvant concurrent chemoradiotherapy? PLoS One. 2014; 9:e88598.
9. Chandarana H, Rosenkrantz AB, Mussi TC, Kim S, Ahmad AA, Raj SD, McMenamy J, Melamed J, Babb JS, Kiefer B, Kiraly AP. Histogram analysis of whole-lesion enhancement in differentiating clear cell from papillary subtype of renal cell cancer. Radiology. 2012; 265:790-798.
10. Haralick RM, Shanmugam K, Dinstein I. Textural features for image classification. IEEE Trans Syst Man Cybern. 1973; 3:610–621.
11. Hung JJ, Yeh YC, Jeng WJ, Wu KJ, Huang BS, Wu YC, Chou TY, Hsu WH. Predictive value of the international association for the study of lung cancer/American Thoracic Society/European Respiratory Society classification of lung adenocarcinoma in tumor recurrence and patient survival. J Clin Oncol. 2014; 32:2357-2364.
12. Eliazar I, Sokolov IM. Maximization of statistical heterogeneity: From Shannon’s entropy to Gini’s index. Physica A: Statistical Mechanics and its Applications. 2010; 389:3023-3038.
13. Son JY, Lee HY, Lee KS, Kim JH, Han J, Jeong JY, Kwon OJ, Shim YM. Quantitative CT analysis of pulmonary ground-glass opacity nodules for the distinction of invasive adenocarcinoma from pre-invasive or minimally invasive adenocarcinoma. PLoS One. 2014; 9:e104066.

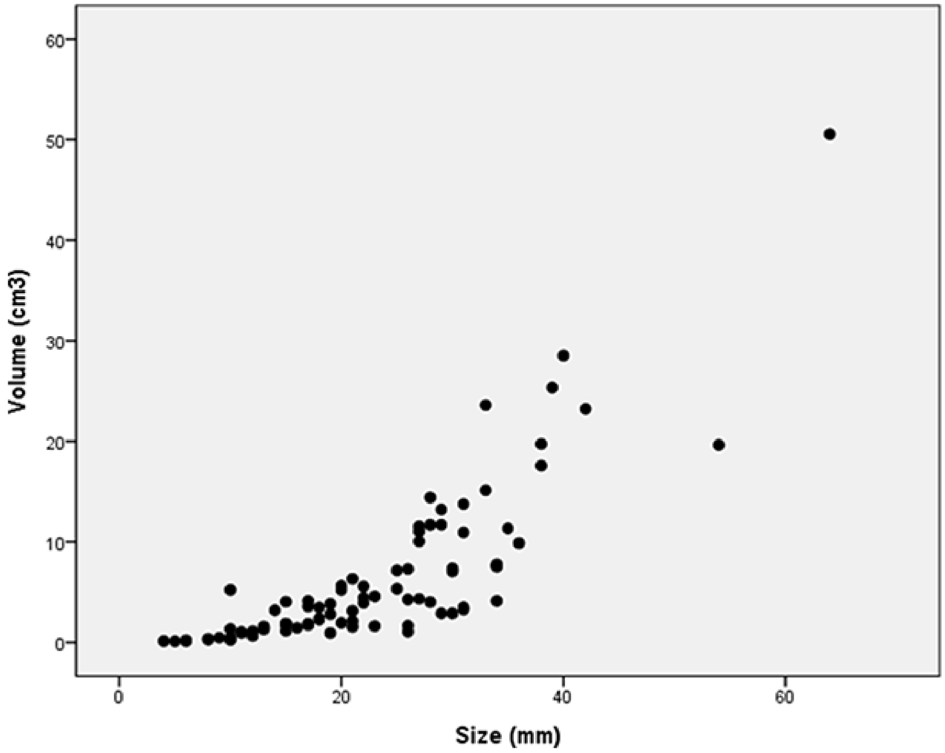

Supplementary Figure S1: The relationship between tumor size and tumor volume.

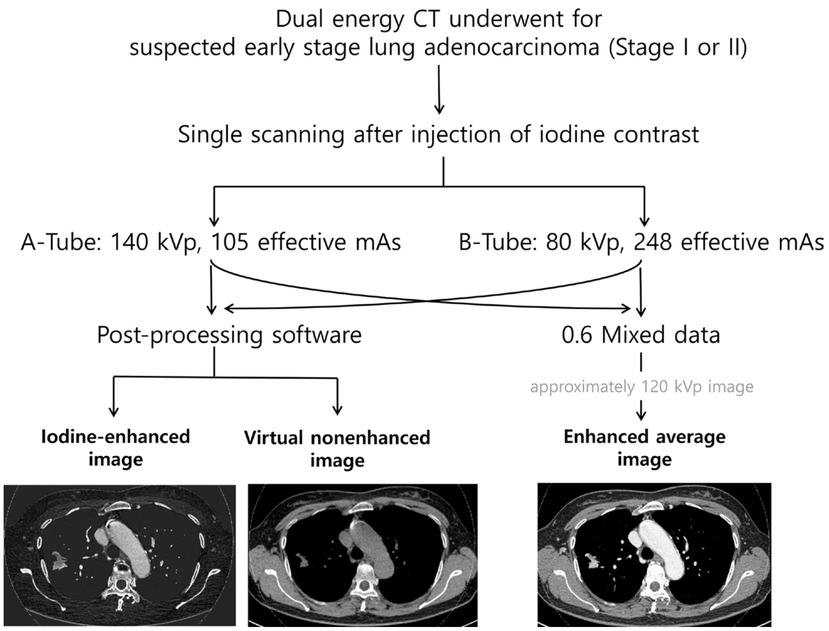

Supplementary Figure S2: Flow of imaging acquisition and reconstruction from dual energy CT.

Supplementary Table S1: The mathematical formulations of quantitative radiomic features

See Supplementary File 1
