# Supplementary material for: Quantitative image variables reflect the intratumoral pathologic heterogeneity of lung adenocarcinoma
Source: Oncotarget. 2016 Aug 30;7(41):67302–13. doi: 10.18632/oncotarget.11693 (PMC5341876; doi:10.18632/oncotarget.11693)
Supplement: Supplementary file 2 [file oncotarget-07-67302-s002.docx]

**Supplementary Table 1:** The mathematical formulations of quantitative radiomic features

|  | **Parameter** | **Formula** | | **Description** |
| --- | --- | --- | --- | --- |
| **Physical feature** | Volume | $volume=R*number of voxels$ [[3](#_ENREF_3)]  Where $R$ denote the 3d image resolution | | Volume of tumor (ROI) |
|  | Density | N/A | |  |
|  | Mass | $mass=V*D$ [[13](#_ENREF_13)]  Where $V$ denote the tumor volume, $D$ denote the tumor density | | Mass of tumor (ROI) |
|  | Size | N/A | |  |
| **Histogram based feature** | Skewness | $skewness=\frac{E{(X-\mu)}^{3}}{\sigma^{3}}$ [[3](#_ENREF_3)]Where $\mu$ denote the mean of$X$, $\sigma$ denote the standard deviation of$x$, $E$ denote the expectation operator. | | Measures asymmetry of a histogram. |
|  | Kurtosis | $kurtosis=\frac{{E(X-\mu)}^{4}}{\sigma^{4}}$ [[3](#_ENREF_3)]  Where $\mu$ denote the mean of$X$, $\sigma$ denote the standard deviation of$x$, $E$ denote the expectation operator. | | Measures “peakedeness” of a histogram (flatness of histogram) |
|  | Histogram percentile | $percentile= \frac{n}{100}\times X$  Where $n$ denote the percentage (2.5, 25, 50, 75, 97.5), $X$ denote the 3d image matrix with $N$ voxel. | | Measures average of a positive histogram value. |
| **Regional features** | Uniformity | $uniformity=\sum_{i=1}^{N_{l}} {P(i)}^{2}$ [[3](#_ENREF_3)]  Where $P$ denote the first order histogram with $N_{l}$ discrete intensity levels. | | Measures HU at the n^th^ percentile on histogram |
|  | Entropy | $entropy=-\sum_{i=1}^{N_{l}} P(i)\log_{2} P(i)$ [[3](#_ENREF_3)]  Where $P$ denote the first order histogram with $N_{l}$ discrete intensity levels. | | Measures uniformity of a histogram. |
|  | Size-zone variability | size-zone variability = $\frac{1}{\Theta}{\sum_{m=1}^{M} \left[ \sum_{n=1}^{N} \boldsymbol{P}\left( m,n \right) \right]}^{2}$[[13](#_ENREF_13)] | | Variability in size of ROI |
|  | Intensity variability | intensity variability = $\frac{1}{\Theta}{\sum_{n=1}^{N} \left[ \sum_{m=1}^{M} \boldsymbol{P}\left( m,n \right) \right]}^{2}$[[13](#_ENREF_13)] | | Variability in the intensity of ROI |
|  | Where $\boldsymbol{P}\left( m,n \right)$ denote the intensity size zone matrix  $\Theta$ represents the number of homogeneous areas in tumor,  $M$ denote the number of distinct intensity values,  $N$ denote the size of homogeneous area in the matrix $\boldsymbol{P}\left( m,n \right)$ | | | |
| **Local feature** | Energy | $energy=\sum_{i=1}^{N_{g}} \sum_{j=1}^{N_{g}} \left[ \boldsymbol{P}\left( i,j \right) \right]^{2}$ [[10](#_ENREF_10)] |  | |
|  | Entropy | $entropy=-\sum_{i=1}^{N_{g}} \sum_{j=1}^{N_{g}} \boldsymbol{P}(i,j)\log_{2} [\boldsymbol{P}\left( i,j \right)]$ [[10](#_ENREF_10)] | Measures irregularity of gray level. | |
|  | Correlation | $correlation=\frac{\sum_{i=1}^{N_{g}} \sum_{j=1}^{N_{g}} ij\boldsymbol{P}\left( i,j \right)-\mu_{i}\left( i \right)\mu_{j}(j)}{\sigma_{x}\left( i \right)\sigma_{y}(j)}$ [[10](#_ENREF_10)] | Measures gray tone linear-dependencies of the gray level | |
|  | Contrast | $contrast=\sum_{i=1}^{N_{g}} \sum_{j=1}^{N_{g}} \left\vert i-j \right\vert^{2}\boldsymbol{P}(i,j)$ [[10](#_ENREF_10)] |  | |
|  | Variance | $variance=\sum_{i=1}^{N_{g}} \sum_{j=1}^{N_{g}} \left( i-\mu\right)^{2}\boldsymbol{P}\left( i,j \right)$ [[10](#_ENREF_10)] |  | |
|  | Sum mean | $sum mean=\sum_{i=1}^{{2N}_{g}} \boldsymbol{[i}\boldsymbol{P}_{\boldsymbol{x+y}}\left( \boldsymbol{i} \right)\boldsymbol{]}$ [[10](#_ENREF_10)] |  | |
|  | Inertia | $inertia=\sum_{i=1}^{N_{g}} \sum_{j=1}^{N_{g}} \left( i-j \right)^{2}\boldsymbol{P}\left( i,j \right)$ [[3](#_ENREF_3)] |  | |
|  | Cluster shade | $cluster shade=$  $\sum_{i=1}^{N_{g}} \sum_{j=1}^{N_{g}} \left[ i+j-\mu_{x}\left( i \right)-\mu_{y}\left( j \right) \right]^{3}\boldsymbol{P}(i,j)$ [[3](#_ENREF_3)] |  | |
|  | Cluster tendency | cluster tendency =  $\sum_{i=1}^{N_{g}} \sum_{j=1}^{N_{g}} \left[ i+j-\mu_{x}\left( i \right)-\mu_{y}\left( j \right) \right]^{2}\boldsymbol{P}(i,j)$ [[3](#_ENREF_3)] |  | |
|  | Homogeneity | $homogeneity=\sum_{i=1}^{N_{g}} \sum_{j=1}^{N_{g}} \frac{\boldsymbol{P}\left( i,j \right)}{1+\left\vert i-j \right\vert}$ [[3](#_ENREF_3)] | Measures closeness of gray-level. | |
|  | Maximum probability | $maximum probability=max\{\boldsymbol{P}\left( i,j \right)\}$ [[3](#_ENREF_3)] |  | |
|  | Inverse variance | $inverse variance=\sum_{i=1}^{N_{g}} \sum_{j=1}^{N_{g}} \frac{\boldsymbol{P}\left( i,j \right)}{\left\vert i-j \right\vert^{2}},i\neq j$ [[3](#_ENREF_3)] |  | |
|  | Where $\mathbf{P}\left( i,j \right)$denote the gray level co-occurrence matrix for ($\delta=1, \alpha=0)$,  $N_{g}$denote the number of discrete intensity value in the image,  $\mu$ denote the mean of $\mathbf{P}\left( i,j \right),$  $p_{x}\left( i \right)=\sum_{j=1}^{N_{g}} \boldsymbol{P}(i,j)$ denote the marginal row probabilities,  $p_{y}\left( i \right)=\sum_{i=1}^{N_{g}} \boldsymbol{P}(i,j)$ denote the marginal column probabilities,  $\mu_{x}$ denote the mean of $p_{x}$,  $\mu_{y}$ denote the mean of $p_{y}$,  $\sigma_{x}$ denote the standard deviation of $p_{x}$,  $\sigma_{y}$ denote the standard deviation of $p_{y}$,  $p_{x+y}\left( k \right)=\sum_{i=1}^{N_{g}} \sum_{j=1}^{N_{g}} \boldsymbol{P}\left( i,j \right) , i+j=k, k=2,3,\ldots,2N_{g}$,  $p_{x-y}\left( k \right)=\sum_{i=1}^{N_{g}} \sum_{j=1}^{N_{g}} \boldsymbol{P}\left( i,j \right) ,\left\vert i-j \right\vert=k, k=0,1,\ldots,N_{g}-1$,  $HX=-\sum_{i=1}^{N_{g}} \boldsymbol{P}_{x}(i)\log_{2} \left[ p_{x}(i) \right]$ denote the entropy of $\boldsymbol{P}_{x}$,  $HY=-\sum_{i=1}^{N_{g}} \boldsymbol{P}_{y}(i)\log_{2} \left[ p_{y}(i) \right]$ denote the entropy of $\boldsymbol{P}_{y}$,  $H=\sum_{i=1}^{N_{g}} \sum_{j=1}^{N_{g}} \boldsymbol{P}\left( i,j \right)\log_{2} \left[ \boldsymbol{P}(i,j) \right]$denote the entropy of $\boldsymbol{P}\left( i,j \right)$  $HXY=-\sum_{i=1}^{N_{g}} \sum_{j=1}^{N_{g}} \boldsymbol{P}\left( i,j \right)\log(p_{x}\left( i \right)p_{y}\left( j \right))$,  $HXY=-\sum_{i=1}^{N_{g}} \sum_{j=1}^{N_{g}} p_{x}(i)p_{y}(j)\log(p_{x}\left( i \right)p_{y}\left( j \right))$. | | | |
